# Decoding Circadian Rhythm and Epileptic Activities: Clues From Animal Studies

**DOI:** 10.3389/fneur.2020.00751

**Published:** 2020-07-24

**Authors:** Cenglin Xu, Jie Yu, Yeping Ruan, Yi Wang, Zhong Chen

**Affiliations:** ^1^College of Pharmaceutical Science, Zhejiang Chinese Medical University, Hangzhou, China; ^2^NHC and CAMS Key Laboratory of Medical Neurobiology, College of Pharmaceutical Sciences, Institute of Pharmacology and Toxicology, Zhejiang University, Hangzhou, China

**Keywords:** circadian rhythm, sleep, epilepsy, animal model, mechanism

## Abstract

The relationship between circadian rhythm and epilepsy has been recognized for decades. Yet many questions underlying the complex mechanisms of their interaction remain elusive. A better understanding on this topic allows the development of accurate seizure-detection algorithm and alternative precise therapeutic strategies. Preclinical laboratory studies based on epileptic animal models, with controllable epileptogenic pathology and an array of intervention strategies, shed light on the bidirectional effects between circadian rhythm and epileptic seizures as well as their underlying mechanisms. Here, we reviewed findings on the interaction between circadian rhythm and epileptic seizures in the preclinical setting. We present the possible mechanisms at molecular, cellular and circuitry levels. We propose that future experimental designs should take into account the relationship between circadian rhythm and epilepsy as well as the underlying mechanisms in different types of animal models, which may have a translational significance as stepping stones for clinical benefits.

## Introduction

Circadian rhythm is one of the most basic rhythms in every organism, giving rise to our night-and-day variations in vital activities, especially mammals. The circadian rhythm can both physiologically and pathologically affect the brain in multiple aspects, among which the regulation of sleep/wake cycle (1–3) is considered the most important. Physiologically, the maintenance of sleep/wake rhythm, especially the ultradian rapid eye movement/non-rapid eye movement (REM/NREM) sleep cycle, is important for various daily activities in human beings. Pathologically, especially in epilepsy, the close relationship between circadian rhythm and epileptic activities has been recognized for years and emerges with an ever-increasing importance (4). On the one hand, epileptic activities are usually presented in a circadian pattern (5). A better understanding of the pattern would contribute to an accurate prediction of seizure onset and the development of closed-loop treatments in the future (6, 7). On the other, the epileptogenic pathology might interfere with the normal circadian rhythm especially the sleep/wake cycle, thereby exerting a large impact on people's daily living and even resulting in severe consequences (8). Despite the great progress have been already achieved through clinical studies, our knowledge regarding the underlying mechanism of circadian rhythm and epilepsy remained far from adequate, mainly limited by the confounding factors in observational studies (such as genetic variations, anti-epileptic medications, and psychiatry comorbidities) (4). The limitation, however, could be well-circumvented by bench research using epileptic animal models and multifaceted intervention strategies, which provides valuable insights into the underlying mechanisms (9). In this review, we describe how did the circadian rhythm and epileptic activities influence each other based on evidence from laboratory studies employing different epileptic animal models. We summarize the possible mechanisms at the molecular, cellular and circuitry level. Lastly, the perspective with a translational insight are provided for a greater basic-clinical integration in the future.

## Sleep Activity and Circadian Rhythm-Dependent Epileptic Activities

The circadian timing system in the human body regulates the timing of the sleep-wake cycle and modulates brain activities (10). Physiologically, the dynamic excitation-inhibition balance of brain network as well as the secretion of hormones such as cortisol or melatonin follows a circadian rhythm are both related with the sleep-wake cycle. Similarly, the epileptic brain also exhibits a day-and-night variation in its excitation-inhibition balance, due to the different brain excitability in the active (mainly wakefulness) and inactive (mainly sleep) phases (4, 11).

### Sleep Activity Influences Seizure Susceptibility in Animal Models

#### Kindling Models

In animal studies, the different seizure susceptibility in sleep and wakefulness was first studied in kindling models. In 1982, Calvo et al. reported that rats receiving amygdaloid kindling stimulations during REM sleep had a delayed progression of kindling process than those receiving kindling stimulations during wakefulness (12). Another study examining the seizure susceptibility in light/dark phases also showed that electric induced seizure thresholds were higher in the dark phase compared with those in the light phase (13). This is further verified by Kumar et al., who showed that increasing the REM sleep resulted in a decreased seizure susceptibility in kindled rats (14). The influence of sleep activities on kindled seizure susceptibility was further studied using paradoxical sleep deprivation (PSD). In the 1980s, Shouse and his colleagues performed a series of experiments on amygdaloid kindled temporal lobe epilepsy (TLE) cats to study the effect of PSD on epileptic seizures. They found that the loss of both REM and NREM sleep caused by PSD could increase the seizure susceptibility (15–17).

#### Other Epileptic Models

The effect of sleep on seizure susceptibility is also verified on acute models. In 1988, Vale and Leite performed PSD on mice. Similar to kindled animals, this manipulation increased the susceptibility to pentylenetetrazol-induced acute seizures (18). PSD could also increase seizure activities in a genetically epileptic WAG/Rij rats. The number of spike-wave discharges increased during the early period of the 12 h-long PSD, and then returned to the baseline level (19).

Taken together, based on existing studies in different models, it has been shown that sleep patterns can directly modulate the susceptibility of seizures. Sleep deprivation, although may induce other many complex physiological changes, could increase seizure susceptibilities.

### Epileptic Seizures Occur With Circadian Rhythmicity in Chronic Models

#### Chronic Temporal Lobe Epileptic Models

**Figure 1:**
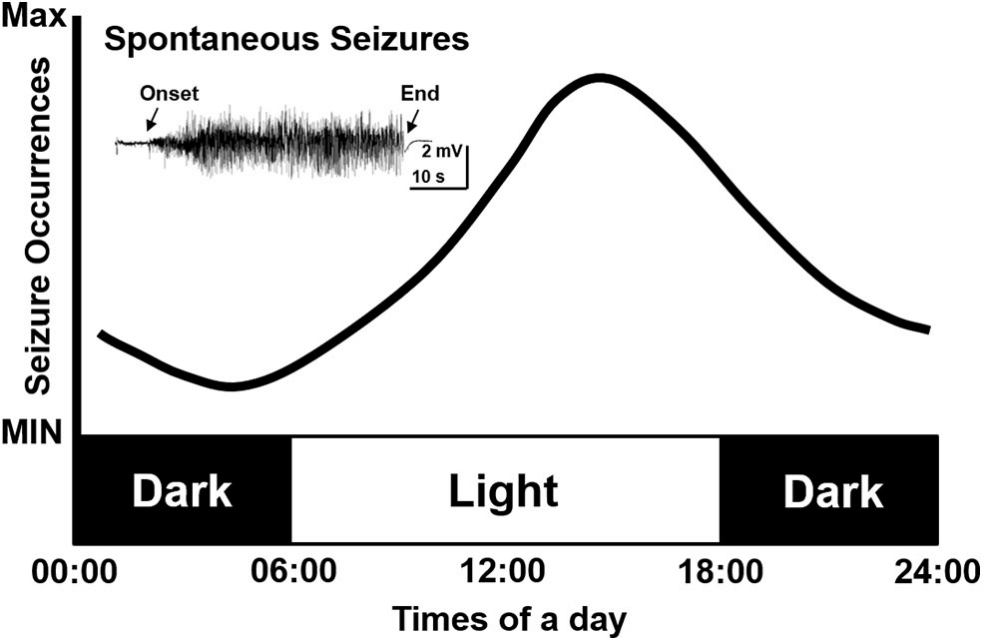
Simulative circadian distribution of spontaneous recurrent seizures in chronic TLE models. The distribution of spontaneous recurrent seizures is not totally random in different phases but shows a circadian pattern in epileptic rodents. The seizures occur more often in the light phase.

As clinical studies suggested, most seizures occur in a temporal pattern following the circadian rhythm (5, 20, 21). To further study the effect of circadian rhythm on epileptic seizures, chronic models of epilepsy were used. Unlike the models of acute seizures (9, 22), chronic models usually exhibit spontaneous recurrent seizures (SRS) after experiencing chemical convulsant or electrical stimulation induced status epilepticus (SE), which mimics the seizure presentation in patients (23–25). With the advent of digital technology, the development of long-term EEG recording contributed to an ever deeper understanding of the circadian pattern of epileptic seizures in chronic models of epilepsy. Relevant studies were also consecutively reported since the 1990s. Cavalheiro et al. reported that rats experiencing pilocarpine induced SE showed a higher seizure frequency in the light phase (26). Following that, a comparison study performed by Quigg et al. also demonstrated that seizures occurred more often during light phase in both rats and humans (27). The circadian distribution of spontaneous seizures was further reported in kainate acid or electrical stimulus induced chronic epileptic rats. Also in those studies, a higher possibility of seizure occurrence in the light phase were presented as well (28–32). Another study by Pitsch et al. further revealed a striking clustering of spontaneous seizures at the transition from the light to dark period in pilocarpine treated mice (33). Even after light deprivation, the occurrence of seizures still showed a circadian pattern (2). However, contradictory results exist as well, some studies reported that seizure frequency in pilocarpine-treated rats was unrelated to the circadian rhythm. The discrepancy might be due to the different age of animals used in different studies (34). Till now, we may conclude that the majority of findings, mostly from chronic TLE models, support a circadian distribution of seizures. Furthermore, seizure activities are more prominent in the light phase, which is the inactive period of rodents (Figure 1).

#### Other Chronic Epileptic Models

Apart from the classic chronic TLE models, experiments were carried out in other types of epileptic models which exhibited SRS as well (35, 36). Interestingly, the findings were contradictory to those in TLE models. Just as Stewart et al. proposed, in an atypical absence epilepsy model where absence seizures originate from the neocortex, the duration of seizure-like discharges reached two peaks at the onset of dark and light phase (35). The same group further studied this issue in an *Aldh5a1* gene deficient epileptic mice. It is a gene encoding Mitochondrial NAD^+^-dependent succinate semialdehyde dehydrogenase [SSADH] enzyme that catalyzes the last step of the GABA shunt pathway (37, 38). In this study, genetically deficient mice showed generalized tonic-clonic seizures in their early life, and the seizure rhythm in the *Aldh5a1*^−/−^ mice occurred with a 24 h periodicity and reached the peak during the early dark phase (36). So far, no direct evidence explained the discrepancy of circadian seizure occurrence between different animal models of epilepsy. The distinct locations of seizure onset zones may contribute to this and emerges as an interesting issue to study on in the future.

To conclude, cumulative results supported a circadian variation of seizure occurrence or seizure susceptibility in epileptic animals throughout the day. However, contradictory findings are found in different animal models, the different types of epilepsy and seizure onset zones would influence the circadian pattern of seizures (39–42). Chronic TLE animals usually display a higher chance of seizure occurrence in the light phase, videlicet, the inactive period (Table 1). However, it has been reported that seizure occurrence was mainly in relation to wakefulness for TLE patients (43, 44). The possible reasons to explain this discrepancy may be the differences in the causes of epileptogenesis and brain circuits between human and rodents. However, both results from TLE animals and patients revealed the circadian distribution of seizures, which suggest those models as useful tools for translational studies aimed at seizure prediction based on circadian rhythm or the development of chronotherapies. Unlike classic chronic TLE models, in an *Aldh5a1*^−/−^ knock out mice which showed spontaneous motor seizures, the spontaneous seizure activities occurred daily in a non-random pattern and reached two peaks at the onset of dark and light phase (36). Although a few studies focused on other types of epilepsy, more efforts are needed to study the circadian pattern of seizures in other types of animal models such as neocortical or frontal epilepsy which are also common types in clinical practice.

**Table 1:** Summary of circadian epileptic seizures in chronic TLE models.

**Studies**	**Model**	**Animals**	**Circadian** **seizures (Y/N)**	**Seizure occurs** **prominently in** **which phase**
Cavalheiro et al. (26)	Pilocarpine	Rat	Yes	Light phase
Quigg et al. (27)	Electric stimulus	Rat	Yes	Light phase
Hellier and Dudek (31)	Kainate acid	Rat	Yes	Light phase
Quigg et al. (28)	Electric stimulus	Rat	Yes	Light phase
Raedt et al. (32)	Kainate acid	Rats	Yes	Light phase
Tchekalarova and Pechlivanova (29)	Kainate acid	Rat	Yes	Light phase
Bajorat et al. (34)	Pilocarpine	Rat	No	N/A
Sedigh-Sarvestani et al. (30)	Tetanus toxin	Rat	Yes	Light phase
Pitsch et al. (33)	Pilocarpine	Mice	Yes	Transition from light to dark phase

## Epilepsy Influences Sleep and Circadian Rhythm in Animal Models

### Epilepsy Influences Sleep in Kindling Models

Epileptic seizures could also in turn alter sleep cycle or even circadian rhythm under the influence of epileptogenic pathology. Many studies provided evidence on the altered sleep activities caused by epileptic seizures. The influence of epileptic seizures on sleep-wakefulness was first reported in kindling models of epilepsy. Since 1980s, studies performed on kindling rats or cats reported that REM sleep activities were inhibited by electrical-stimulation induced seizures (45–47). In 1998, however, Raol and Meti performed a systematic study about sleep activities in amygdaloid kindled rats and reported contradictory results. They found that stage 5 seizures could increase the duration of deep slow-wave sleep (SWS) and decrease the duration of both the NREM sleep and wakefulness (48). The discrepancy might be due to the different time of day when stimulations were given, just as Yi et al.'s reported on amygdala kindling seizures. Kindling stimulation given at the beginning of the light phase decreased both SWS and REM sleep, while kindling stimulation given at the dark phase increased SWS but had no effect on REM sleep (49).

### Epilepsy Influences Sleep in Chronic TLE Model

In chronic TLE models, indisputable results have presented the altered sleep activities. By analyzing 6 h long (6–12 a.m.) sleep architecture in rats 15 weeks after electrical stimulation induced SE. Bastlund et al. found an increase in the average time spent in wakefulness, and a decrease in the time spent in paradoxical sleep (50). A later study further analyzed distribution of sleep-wake phases during 24 h in pilocarpine treated rats. The epileptic rats showed reduction of attentive wakefulness concomitant with increased SWS (51).

### Epilepsy Influences Sleep in Genetic Epileptic Model

The altered sleep architecture is also found in several genetic epileptic models. Just as in WAG/Rij rats with spontaneous absence seizures, like TLE models, both NREM sleep and the sleep cycle were disrupted by epileptic activities (52). In *Kcna1*-null mice with SRS, durations of both NREM and REM sleep was less than those in WT mice (53). Meanwhile, in a mouse genetical mouse model of Dravet syndrome (DS), a rare epilepsy syndrome, Kalume et al. reported sleep impairment in DS mice as well, including reduced delta power and sleep spindles and increased brief wakefulness in NREM sleep (54).

In brief, epileptic activities undoubtedly influence the architecture of both sleep and wakefulness. Different animal models may show contradictory results, probably due to the diverse brain regions involved in epileptogenesis, which may affect the circadian activities in different ways. The data from animal studies reproduce many relevant features observed in clinical patients and thus highlight the role of these models in studying sleep dysfunction in epilepsy.

### Epilepsy Influences Other Type of Circadian Rhythm

Besides sleep activities, the influence of epileptic seizures on other circadian activities has also been reported. In 2001, by using radio telemetry as a measurement of the temperature on hippocampal kindled rats, Quigg et al. demonstrated that electrically-induced seizures shifted the circadian temperature rhythms (CRT) which was different from the typical light-induced phase shifts. In their study, the CRT in the first postictal 24 h were more complex and polyrhythmic than preictal conditions (55). As proposed by Smith et al., seizures could also affect the amplitude but not phase of the circadian clock. They applied a maximal electroconvulsive stimulation at different time-points of the day on hamsters. To their surprise, only the level of circadian locomotor activity but not the phase were significantly attenuated (56). Pilocarpine induced SE has also been reported to influence circadian rhythms. In mice treated by pilocarpine, the circadian EEG was transiently suppressed for several days after SE (33). A recent study further reported altered circadian rhythms in a model of sudden unexpected death in epilepsy. In this study, using passive infrared actigraphy to access circadian rest-activity patterns in epileptic rodents, Wallace et al. found disrupted diurnal and circadian rest-activity patterns characterized by prolonged circadian periods (53).

To conclude, animal models could reliably show an altered circadian rhythm and sleep activities as induced by epileptic seizures. The similarity to clinical conditions demonstrates that these models can be used to study the sleep disturbance and the disrupted circadian rhythm in epilepsy. Potential treatments for epilepsy-related sleeping disorders can be tested with these models. Valuable data obtained from these animal models would provide adequate grounding to help epileptic patients who suffer from sleep disorders as comorbidities.

## Epilepsy-Induced Changes in Circadian Rhythm and Sleep Architecture: The Possible Mechanisms

### Altered Oscillatory Expression of Rhythm-Related Molecules in Epileptic Animal Models

“Clock” proteins including BMAL (brain and muscle, ARNT-like)1 and CLOCK (circadian locomotor output cycles kaput) in the hypothalamic suprachiasmatic nucleus (SCN) are autoregulators of circadian rhythm in mammals (57, 58). The feedback loops of these “clock” proteins lead to the establishment of circadian rhythms. For example, CLOCK and BMAL1 form heterodimers activate the transcription of downstream genes such as Period (Per1) and Cryptochrome (Cry1). BMAL1 reaches its peak of expression at mid-night, while the transcription of Pers and Crys (anti-phase to BMAL1 expression) reach their peaks during mid to late day. Pers and Crys along with other proteins form heteromultimeric complexes and directly abrogate the transcriptional activity of the CLOCK-BMAL1 complex, further leading to lowered Pers and Crys mRNA levels. This feedback loop along with others is the most important components of physiological day-and-night clock (59).

**Figure 2:**
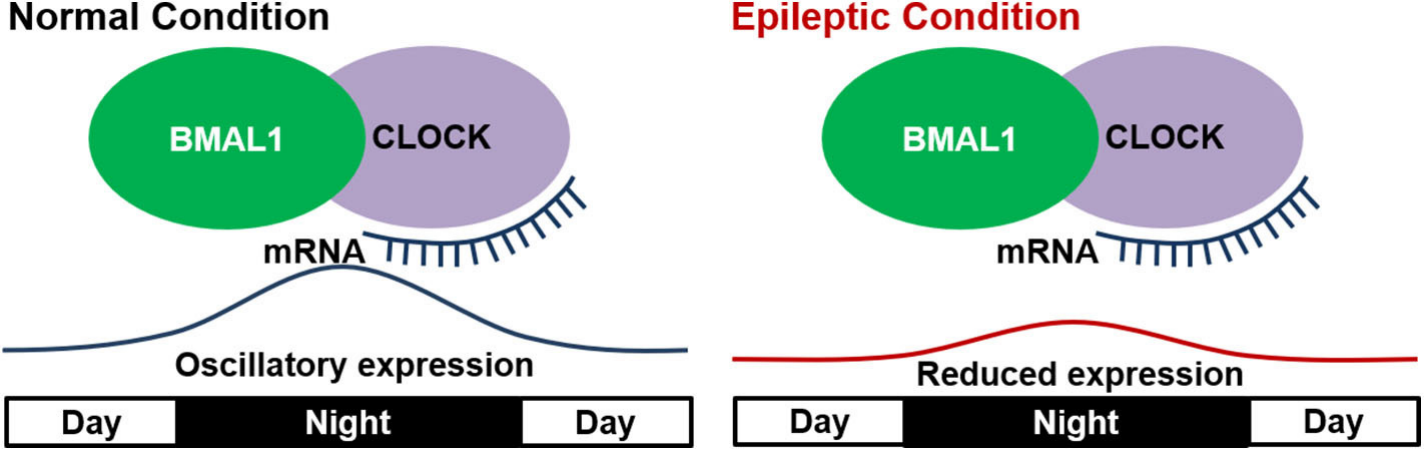
Disrupted expression of “clock” genes in epilepsy. In the normal condition, “clock” genes including BMAL1 and CLOCK show a diurnal oscillatory expression with a peak at mid night. In epileptic condition, the oscillatory expression of “clock” genes is reduced and thus leads to altered circadian rhythm.

Several studies showed that mutations or deletion in these core proteins could impact circadian rhythms (60–62). In epilepsy, a study based on kindled mice showed that BMAL1 knock-out not only eliminated the circadian difference of seizure susceptibility, but also directly lowered the seizure threshold (13). These results suggested the involvement of “clock” proteins in circadian epileptic activities and even epileptogenesis. Other studies on the oscillatory expressions of clock genes in epileptic animals further revealed its importance in a dynamic manner. In *Kcna1* knockout epileptic mice, ketogenic diet was shown to improve the disturbed diurnal rhythmicity (63), which may be due to the circadian clock's phase shift mediated by *SirT1* (a key factor involved in metabolism and life span, which interacts directly with CLOCK genes) (64). Further studies by Wallace et al. first provided direct evidence on the relationship of clock proteins and changed circadian rhythm in epileptic *Kcna1-null* mice. In their studies, all the epileptic mice manifested disrupted diurnal and circadian rest-activity patterns, and showed a reduced oscillatory expression of several clock proteins (*Clock, Per1*, and *Per2*) and diurnal *Sirt1* mRNA in the anterior hypothalamus (53). The abnormal circadian rhythms in other neurogenerative diseases were also found to be closely related with an attenuation of oscillatory expressions of clock genes (65–67). Thus, the altered oscillation of clock genes is connected with altered circadian rhythms in epilepsy (Figure 2). However, whether the conclusion can be generalized to epilepsies caused by various factors and pathologic processes needs further validation.

Besides “clock” genes, there are other molecules in the mammals' brain regulating circadian rhythms through a circadian pattern of expression. Study of the 24 h expression pattern of these molecules in epileptic animal models might provide clues to the circadian epileptic activities. Retinoic acid receptor-related orphan receptor alpha (RORα) is a member of a nuclear receptor superfamily and plays an essential role in regulating the circadian rhythm (68). In mammals, the RORα is a crucial molecule in the regulation of BMAL1 expression. In hypothalamic SCN, RORα shows an oscillatory expression, leading to the circadian pattern of BMAL1 expression (69), thereby directly influencing the circadian rhythm. In 2015, Rocha et al. attempted to study the daily pattern of RORα expression in chronic epileptic rats. They found that pilocarpine induced SE could decrease the mRNA expression of RORα in the hippocampal area at both the light and dark phases, whether it be the acute and the silence (no SRS is present in animals) period, or at the 3 h point post the lightning-off in the chronic period (70). The evidence provides clues that acute SE could robustly influence the expression of RORα, leading to a permanent alteration in its circadian expression pattern in the chronic period. The change might explain the altered circadian rhythms in epileptic models.

Melatonin is another important molecule synthesized and released in the pineal gland by a circadian manner, reaching a high level at night (71). Melatonin regulates circadian rhythms through an action on its membrane receptors (MT_1_ and MT_2_) (72). Clinical studies have demonstrated that melatonin could alleviate sleep disturbance and reduce circadian alterations in epileptic patients (73–75); Moreover, one study on treatment-naive active epileptic patients showed an increase of melatonin production in epileptic patients and a circadian pattern with a phase difference between patients and normal people (76). Rocha et al. studied the relationship between melatonin receptors and circadian changes in pilocarpine treated rats. They examined the 24 h profile of mRNA and protein expression of MT_1_ and MT_2_ receptor in different phases of pilocarpine models. In the chronic phase during which rats expressed SRS and an altered circadian rhythm, mRNA expression levels of both receptors return to levels close to control, however, presenting a different daily profile (77). In detail, MT1 receptor mRNA expression only decreased in the zeitgeber time point (ZT) 0 which is the transition phase of the light/dark cycle, and MT2 receptor mRNA expression increased at nighttime (ZT18). These biochemical results in animals revealed an altered expression of circadian rhythm related molecules in the chronic phase of epileptic models, which further suggested a close relationship between these molecules and the altered circadian rhythm in epilepsy. However, systematic experiments employing various intervention strategies along with detailed behavioral recordings are still needed.

### Involvement of Other Non-Rhythmic-Related Molecules in Epileptic Animal Models

Neuroinflammation is widely involved in many neurological conditions including sleeping activities (78). Interleukin-1β (IL-1β), one of the most important neuroinflammatory cytokines, is reported to be closely related with epiletogenesis according to numerous studies (79–82). In the epileptic brain, seizures up-regulated the concentration level of IL-1β and the expression of IL-1 receptors, which further contributed to epileptic seizures or other comorbidities (80). Besides, IL-1β has an divergent effect on NREM sleep with different doses (83, 84). Therefore, IL-1β may mediate the epilepsy-induced sleep disturbances in epileptic patients. Yi et al. firstly revealed the crucial role of IL-1β and IL-1β receptor in epilepsy-induced sleep disruption. They performed kindling stimulation at the onset of dark phase, when SWS was induced along with an increased IL-1β mRNA expression in the brain. Administration of IL-1β receptor antagonist (IL-1ra) blocked the SWS induced by kindling (49). The involvement of IL-1β in an altered sleep architecture in epilepsy is further verified by Huang et al. They performed amygdaloid kindling stimulation at the particular ZT13 in both IL-1β receptor type 1 (IL-1R) KO mice and wildtype (WT) mice. In WT mice, kindling stimulation at ZT13 significantly enhanced NREM sleep, contrary to that observed in IL-1R KO mice (85). Yet no difference of seizure susceptibility between WT and KO mice were found. In consideration that changed IL-1β levels could directly affect seizure susceptibility, whether the involvement of IL-1β in epilepsy-induced sleep disruption is caused by its direct effect on seizures needs further investigation.

### Hypothalamic and Thalamic Pathology Underlies Changed Sleep Architecture in Epileptic Models

The ascending arousal system is governed by neural pathways originating from well-defined cell groups in specific regions. The pathway has two branches, the first originating from hypothalamic suprachiasmatic nucleus (SCN), which is the crucial region in the modulation of circadian rhythms (86). Photic inputs are received in the SCN, which further convey the timing information by transcription-translation autoregulatory feedback loop of “clock” proteins, thereby affecting cellular functions including excitability (87). Then the information is outputted through the monoaminergic neurons of hypothalamic SCN to downstream nucleus such as cerebral cortex, which forms the ascending arousal system in the brain (88, 89). Evidence from animals demonstrated that hypothalamus lesions could directly induce long-lasting sleepiness or even somnolence (90, 91). Meanwhile, the endogenous hypothalamic dysfunctions were reported in epilepsy (92–95). Therefore, the hypothalamic pathology in epilepsy might result in disturbances of sleep and circadian rhythms. Sanabria et al. examined light induced Fos-protein expression in the SCN of the hypothalamus in epileptic rats, and the Fos-like immunoreactivity induced by photic stimulation was significantly reduced in chronic epileptic rats (96). Those results indicate that in chronic epileptic rats, light induced response was altered in the hypothalamus, which might reflect altered circadian rhythms. In 1999, Quigg et al. further reported the change of circadian rhythm of temperature (CRT) in epileptic animals, which was associated with a decreased neuronal density in anterior and posterior hypothalamus (97). A further study by Bastlund et al. confirmed the hypothalamic pathology in epileptic rats which showed a changed sleep architecture (50). They revealed that spontaneous epileptic rats following electrical stimulation induced SE showed a high percentage of seizures at sleep, which might be associated with neuronal cell loss in the dorsomedial hypothalamus.

Besides hypothalamic pathway, another ascending arousal pathway is controlled by thalamus. The crucial thalamic nuclei such as the reticular nucleus of the thalamus (RNT) has a role in gating wakefulness (98). Neural activities in some specific regions of the thalamus governs sleep activities (99, 100). Abnormal neural activities caused by epileptogenic pathology might thus result in sleep impairment. In 2015, Kalume et al. uncovered the reduced excitability in RNT interneurons in an epileptic mouse model with DS, and reported its association with sleep impairments (54). In their study, mice with DS, a common childhood-onset epilepsy syndrome, displayed an abnormal sleep architecture depending on activities of inhibitory GABAergic neurons in RNT. Reduced Nav current in the GABAergic RNT neurons led to the reduced rebound neural firing following hyperpolarization, which resulted in the disturbance of sleep architecture.

**Figure 3:**
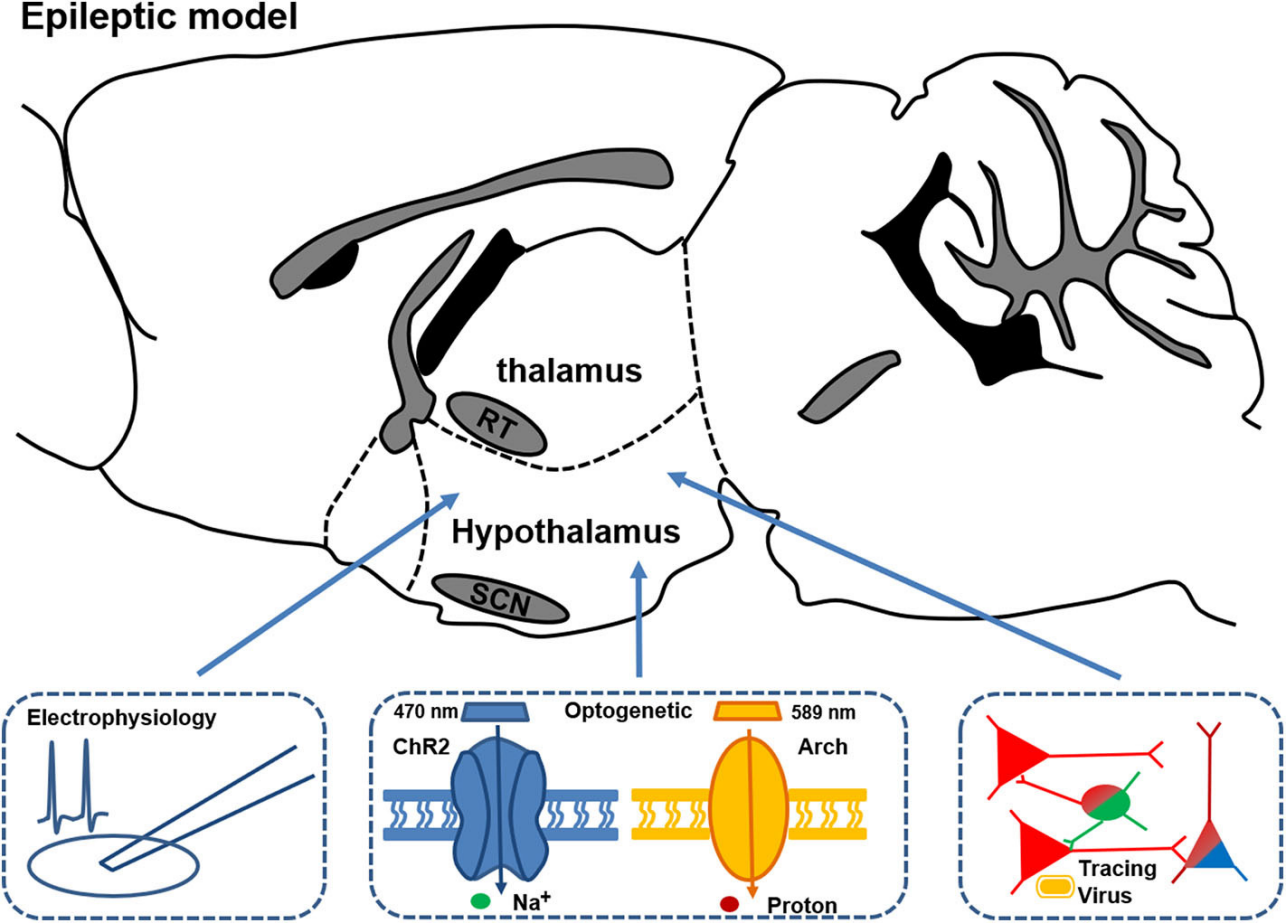
Schematic of the circuitry experiments in thalamus and hypothalamus for studying the mechanisms of changed circadian rhythm and sleep in epileptic models. Combined multifaceted techniques including electrophysiology recording, optogenetics, and viral tracing can help to reveal the possible circuitry mechanism and provide potential precise treatment targets for disrupted circadian rhythm and sleep in epilepsy.

All those previous studies unveiled an evident association between hypothalamus and thalamus abnormalities and the altered circadian rhythm or sleep disturbance in animal models of epilepsy. Nevertheless, how the pathological hypothalamus or thalamus, along with the related neural circuits, contribute to the disturbance in sleep and circadian rhythms requires further investigation (Figure 3). Future studies, with an array of modern techniques, would enable a deeper understanding of the interaction between certain brain regions and circadian rhythm dysfunctions in epilepsy.

## Conclusions and Future Perspectives

Cumulative evidence from animal studies have revealed a close relationship between circadian rhythm and epileptic activities. Evidence from animal studies, using models of chronic TLE, showed an altered temporal distribution of seizures similar to that observed in TLE patients, with a higher frequency of seizures in the light phase. On the other hand, seizure activities would also influence sleep-wake cycles, sleep architecture, and circadian rhythm. It may be attributed to an altered expression of rhythm-related or other non-rhythmic molecules, together with hypothalamus or thalamus pathologies. The advancement of technology and animal models gives animal studies a greater translational value. Therefore, animal models could be harnessed as useful tools to study the relationship between circadian rhythm and epilepsy.

Previously, most studies on the circadian distribution of epileptic seizures were performed on animal models of chronic TLE. More efforts should be spent on accurate seizure predictions based on circadian rhythms and also the development of closed-looped seizure control systems, which is currently studied by some ongoing clinical research (101). Animal studies, with its translational value, can yet provide clues at the molecular, cellular and circuitry level. In particular, epilepsy is gradually considered as a disorder of abnormal neural networks (102). Although accumulating findings provide valuable clues at molecular and cellular levels, molecular and cellular mechanisms are still integrated at the level of brain networks, and the microcosmic perspective might restrict the understanding of epilepsy-induced circadian rhythm dysfunction. Delightfully, accumulating evidence revealed the crucial role of the hypothalamus and thalamus in circadian epileptic activities. Employment of advanced techniques including optogenetics, viral neuronal tracing et al. would allow a deeper understanding of circadian epileptic activities at the circular level. Individualized treatments for epilepsy-related circadian disturbances would also benefit from those animal studies as well.

In sum, animal studies could be well-harnessed when studying the relationship between circadian rhythm and epilepsy. Endeavors to bridge the gap between bench and bedside would greatly assist in the diagnosis, prediction and treatment of circadian epileptic activities.

## Author Contributions

CX and JY wrote the manuscript. YR, YW, and ZC edited the manuscript. All authors contributed to the article and approved the submitted version.

## Conflict of Interest

The authors declare that the research was conducted in the absence of any commercial or financial relationships that could be construed as a potential conflict of interest.
